# Health literacy and the role of social support in different age groups: results of a German cross-sectional survey

**DOI:** 10.1186/s12889-023-17145-x

**Published:** 2023-11-16

**Authors:** Julia Klinger, Eva-Maria Berens, Doris Schaeffer

**Affiliations:** 1 Institute of Sociology and Social Psychology, University of Cologne, 50931 Cologne, Germany; 2 Ethics Committee, Bielefeld University, 33501 Bielefeld, Germany; 3 School of Public Health, Bielefeld University, 33501 Bielefeld, Germany

**Keywords:** Health literacy, Health information management, Social support, Social context, Social practice, Life span, Life course, Vulnerability

## Abstract

**Background:**

Scholars demand more focus on context-related factors of health literacy as the management of health information is seen as a social practice. One prominent factor is social support that is expected to be particularly relevant for persons vulnerable for low health literacy. It was shown that health literacy can differ across the life span and especially older people have been demonstrated to be vulnerable for low health literacy. Therefore, health literacy and the relation of social support on health literacy in different age groups should be investigated.

**Methods:**

In a German nationwide survey 2,151 adults were interviewed face-to-face. General comprehensive health literacy was measured with the HLS_19_-Q47 which differentiates single steps of health information management – access, understand, appraise, and apply. Social support was measured with the Oslo 3 Social Support Scale. Bivariate and multivariate analyses were performed for all respondents and for five age groups.

**Results:**

Health literacy is relatively low in all age groups but particularly low among old-old people (76 + years). Also, the youngest adults (18–29 years) have slightly lower health literacy than middle-aged adults. On average, health literacy is higher among people with higher social support but this association varies between age groups. It tends to be quite strong among younger adults (18–45 years) and young-old persons (65–75 years) but is weak among older middle-aged (46–64 years) and old-old persons. The association also differs between steps of information management. It is stronger for accessing and applying information but there are differences in age groups as well.

**Conclusions:**

Social support is a relevant aspect to improve individuals’ health literacy and therefore should be addressed in interventions. However, it is necessary to differentiate between age groups. While both young adults and particularly old-old persons are challenged by health information management, young adults can strongly profit from social support whereas it can barely compensate the low health literacy of old-old persons. In addition, different challenges in information management steps in different age groups need to be considered when designing health literacy interventions. Thus, target group specific services and programs are needed.

**Supplementary Information:**

The online version contains supplementary material available at 10.1186/s12889-023-17145-x.

## Background

Managing health information has become increasingly important as the (health) information environment is constantly changing and partly becoming more complex while people in Western societies have increasing autonomy but also responsibility regarding their health (choices). Therefore, health literacy (HL) is understood as a critical determinant of health [1, 2]. In the comprehensive definition by Sørensen et al., HL refers to “the knowledge, motivation and competences to access, understand, appraise, and apply health information in order to make judgements and take decisions in everyday life […] to maintain or improve quality of life during life course” [3]. This study will focus on this comprehensive understanding of HL.

Recognizing the importance of HL for health, there is a vast body of international research on HL and its determinants, mostly from an individual perspective, showing that functional literacy skills and socioeconomic factors like education, social status or financial deprivation (ex. [4–11]) affect HL. However, the social-ecological context in which health information are managed (ex. [3]) has received rather limited attention so far and scholars as well as the World Health Organization (WHO) demand more focus on these context-related factors [12–15]. The WHO especially points out that “The degree to which individuals have responsibility for and take action to access, understand, appraise, remember and use health information and services depends largely on the family, social communities and networks, neighbourhoods and the community or society in which they live. Thus, health literacy develops through people’s daily activities and social interactions where ideas and information about health and health care are exchanged.” ([2] p. 7).

An important part of the social context is social support. The term “social support” is often used in a broad sense and is mostly understood as a psycho-social resource that is accessible in the context of one’s social network and interpersonal contacts [16]. An often neglected but in the light of HL very important aspect of social support is informational support, that is “having a wide range of network ties also provides multiple sources of information and thereby increases the probability of having access to an appropriate information source. […] For example, network members could provide information regarding access to medical services or regarding the benefits of behaviours that positively influence health and well-being.” ([16] p. 11).

In that sense, social support might be relevant as a means to access, understand, appraise and apply health information. Lee et al. [17] pointed out the relevance of social support for HL early on. It is also included as one dimension of HL in the Health Literacy Questionnaire (HLQ [18]). Studies from Australia, Germany and the Netherlands using the HLQ showed that the dimension “Social support for health” is positively correlated with the other HL dimensions [18–20]. The existing studies using a *comprehensive* HL measure show that people who have more social support from relatives or friends are more likely to have higher HL [21–23]. Furthermore, qualitative studies support the relevance of social support for HL (ex. [24–27]).

Specifically, social support is expected to be (more) important for people who are vulnerable for low HL and face more difficulties in accessing, processing and applying information [12]. Vulnerable groups can be identified by distinguishing persons by sociodemographic factors such as age. While there is evidence internationally and in Germany that health literacy is particularly low among older age groups [4, 10, 28–32], other studies found no (linear) correlation of HL and age [4, 32–34] and some studies even indicate a positive age effect on HL [9, 35, 36]. Thus, it can be assumed that there is variation in the role of social support for HL across the life span. However, quantitative findings on social support regarding health information in different age groups are rare and inconsistent. Using the HLQ, a Danish study found a higher level in the dimension “Social support for health” among patients younger than 65 years compared to older patients [37] while the finding was opposite in two Australian samples [38, 39] and there was no difference between these age groups in a Spanish [40], a Dutch [20] and a Slovak sample [41]. While the focus on old versus young adults may seek to focus the erosion of social networks and the possible social isolation of old-aged people as a major social problem in Western societies, the role of social support for HL in other age groups should not be neglected. Therefore, there is need to investigate this relation across the life span.

This analysis attempts to fill this gap by analysing the role of social support for HL in different age groups. The following questions are addressed: What is the current status of comprehensive HL in different age groups in Germany? Which role does social support play for HL in general and in different age groups? Are there differences regarding these relations between the single steps of health information management?

## Methods

This study is based on a survey of 2,151 persons of the adult German-speaking resident population of Germany living in private households (population size is 67.5 million persons). The survey was carried out in December 2019 and January 2020, using paper-assisted personal interviews (PAPI). A multi-stage random sampling was combined with quota sampling to recruit respondents [42]. At the first stage, sampling points were randomly selected in all federal states, considering population density in municipalities. In these 558 sampling points, respondents were selected by the interviewers regarding quota for gender in combination with age group, size of household, and educational level. Therefore, the sample characteristics gender, age group, educational level and federal state are distributed according to population statistics. Immigrants are underrepresented.

The study is part of the international HLS_19_ of the Action Network on Measuring Population and Organizational Health Literacy (M-POHL) of the World Health Organization Europe [43]. Comprehensive HL is measured using the German version of the HLS_19_-Q47 with person’s self-assessed difficulties in 47 specific health-related information tasks in the domains health care, disease prevention and health promotion for the information management steps *access* (13 items), *understand* (11 items), *appraise* (12 items) and *apply* (11 items) [42, 43]. The respondents could choose between the answer options “very easy“, “easy“, “difficult“ and “very difficult”. HL scores for the scales general HL (all 47 items) and each information step is calculated as the share of the first two answer options if 80% of the items of the respective scale were answered, which is in accordance with the HLS_19_ procedure. The scores range from 0 to 100 with higher values indicating higher HL.

Social support is measured with the German version of the validated Oslo 3 Social Support Scale (OSS-3 [44, 45]). With the following 3 items, it is a short instrument to assess general social support including emotional and instrumental support:

Oslo 1: How many people are so close to you that you can count on them if you have great personal problems? 1 ‘none’, 2 ‘1–2’, 3 ‘3–5’, 4 ‘5+’;

Oslo 2: How much interest and concern do people show in what you do? 1 ‘none’, 2 ‘little’, 3 ‘uncertain’, 4 ‘some’, 5 ‘a lot’;

Oslo 3: How easy is it to get practical help from neighbours if you should need it? 1 ‘very difficult’, 2 ‘difficult’, 3 ‘possible’, 4 ‘easy’, 5 ‘very easy’.

The answers are combined in a sum score ranging from 3 to 14 with higher values for higher social support. According to previous research this score is used to form categories: poor (3–8), moderate (9–11) and strong social support (11–14) [45, 46].

Age is categorized into the following five groups according to earlier studies [29, 47]: 18–29, 30–45, 46–64, 65–75, 76 years and older. This allows for a comparison of different phases across the life span without being too small-scaled and having enough cases for detailed analyses.

Various sociodemographic variables were included as correlates of HL and social support. The “International Standard Classification of Education 2011” (ISCED-11) is used to determine the level of education (ranging from 0 “no formal education” to 8 “doctorate”) [48]. Due to low case numbers for the two lowest categories (n = 26), they are combined with ISCED level 2 (lower secondary education) and the only respondent in ISCED level 5 was included in the level above. Therefore, educational level is measured on a scale from 1 to 6. Social status is assessed with a German version of the “MacArthur Scale of Subjective Social Status” based on the perceived position in society ranging from 1 to 10 [49, 50]. Financial deprivation is based on perceived difficulties in paying for medications, medical treatments, and monthly bills. Financial deprivation is assumed when 2 or all three questions were answered with “difficult” or “very difficult”. Furthermore, health-related literacy skills are integrated in the multivariate models. Health-related literacy skills, also referred to as functional HL, were assessed with the German version of the internationally validated objective Newest Vital Sign test (NVS [51]), which consists of 6 questions on the ability to read and understand information on a nutrition label. Higher values indicate higher literacy skills.

Bivariate correlations are analysed with ANOVAs. To investigate the age group differences and the relation between HL and social support further it is necessary to control the effects of confounding variables, i.e. variables that influence health literacy as well as social support. Therefore, various ordinary least square (OLS) regression models are calculated. That is, the bivariate relationships are adjusted for the confounding variables gender, educational level, literacy skills, social status and financial deprivation. Age group differences are tested by including dummy variables for each age group in comparison to the age group with the highest scores of general HL as well as each information management step as dependent variables. The adjusted correlation of these with social support as the independent variable, i.e. explanatory factor, are calculated for the total sample as well as for each age group, respectively. To test for linear and non-linear correlations, the social support score is included both as continuous and as categorical variable, the latter as dummy variables for moderate and strong social support in comparison to low social support. Uni- and bivariate analyses for the total sample are based on the data weighted for gender, age and educational level in accordance to federal state and population density to be equivalent to the population structure according to official statistics [42]. In the regression analyses unweighted data is used and listwise case exclusion is applied resulting in restricted samples.

## Results

The mean age of the respondents in the total sample is 50.8 years. 14.0% of the respondents in the sample have poor social support. The majority of respondents (46.4%) indicated moderate social support and another 35.2% indicated strong social support. Detailed information on the sample is shown in Table 1.

**Table 1:** Sample characteristics (weighted data)

	n	%		n	%
**Total**	2,151	100	**Gender**		
**Age**			male	1,056	49.1
*mean (SD), min-max*	50.8 (18.5)	18–92	female	1,089	50.6
18–29 years	362	16.8	Missing	6	0.3
30–45 years	493	22.9	**Educational level**		
46–64 years	683	31.8	low (1)	238	11.1
65–75 years	338	15.8	medium (2–3)	1,263	58.7
76 years and older	252	11.7	high (4–6)	607	28.2
missing	19	0.9	missing	44	2.1
**Social support**			**Health-related literacy skills**		
*mean (SD), min-max*	10.7 (1.96)	3–14	*mean (SD), min-max*	4.40 (1.64)	0–6
poor (3–8)	301	14.0	missing	102	4.5
moderate (9–11)	998	46.4	**Subjective social status**		
strong (12–14)	756	35.2	*mean (SD), min-max*	5.87 (1.58)	1–10
missing	95	4.4	missing	60	2.8
			**Financial deprivation**		
			no	1,583	73.6
			yes	360	16.7
			missing	208	9.7

Measurement: Social support measured by “Oslo 3 Social Support Scale”; educational level categorised following the “International Standard Classification of Education 2011” (ISCED-11); health-related literacy skills measured by “Newest Vital Sign” test; subjective social status measured by “MacArthur Scale of Subjective Social Status”; financial deprivation measured by perceived difficulties in paying for medications, medical treatments, and monthly bills (yes: 2 or all 3 questions answered with “(very) difficult”, no: 0 or 1)

The mean general HL score of the sample is 61.8 (see supplementary Table 1 for more values). On average, the score is lowest in appraising health information (mean 51.3), followed by accessing and applying (mean scores 63.7 and 65.4). Understanding information is perceived the least difficult information managing step with a mean score of 67.4.

### Health literacy in different age groups

**Fig. 1:**
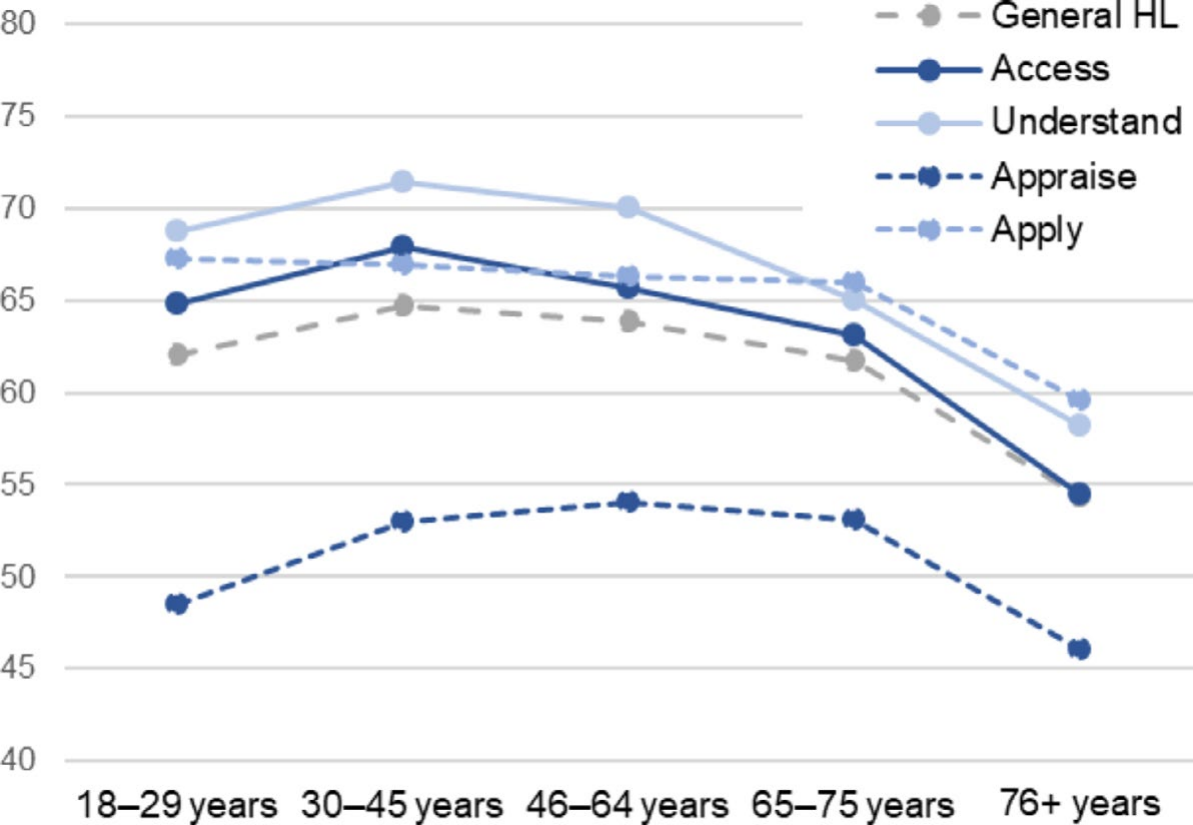
Mean scores of general HL and steps of information management in age groups

First, we investigate HL in different age groups. Respondents in the oldest age group (76 years and older) have considerably lower mean scores than in other age groups, constantly for general HL and in all steps of information management (Fig. 1, values in supplementary Table 1). For general HL, the difference of the mean scores between the oldest age group (54.4) and respondents aged 30–45-years who have the highest score (64.7) is about 10 out of 100 possible points. Their mean scores in accessing and understanding health information are particularly lower than in younger age groups and also significantly lower among respondents aged 65–75 years (63.1 and 65.0). Contrarily, the mean score for appraising information of the youngest adults is significantly lower (48.5) than the scores of participants aged 30 to 75 years. For applying information, there are no substantial variances in the mean scores among all age groups below 76 years. Figure 1 shows that none of the associations are strictly linear but curvilinear.

As differences in the composition of the age groups regarding relevant factors of HL are likely, the variations are tested in regression models that adjust for gender, educational level, literacy skills, social status and financial deprivation. The results are shown in Table 2. For comparison, the unadjusted differences of the restricted sample are included as well. Middle-aged adults (30–45 years) form the reference group as they show the highest general HL on average. The results support the findings of the bivariate analyses. As expected, the differences in the coefficients between the age groups decrease in the adjusted models, indicating that part of the lower HL in the respective age groups are attributed to an unfavourable composition of relevant individual characteristics. After adjusting, the oldest age group (76 + years) still had statistically significantly lower scores than the reference group in all steps of information management except for appraising information, though this coefficient is still relatively high (B=-4.04). Scores for accessing and understanding are particularly lower (B=-9.46/B=-7.6) and those of respondents aged 65–75 years are also still significantly lower than in the reference group after adjusting (B=-3.82/B=-4.9). Regarding appraising information, the youngest age group does not show a statistically significant lower score compared to the reference group but the difference is still somewhat substantial and points to a possible curvilinear association.

**Table 2:** Age group differences of mean scores of general HL and steps of information management

Dependent variable (0–100)	Age group	Unadjusted unstandardized coefficient [CI]	Adjusted^ unstandardized coefficient [CI]
**General HL**	18–29 years	-1.02 [-4.0 – 2.0]	0.447 [-2.5 – 3.3]
	30–45 years	ref.	ref.
	46–64 years	-0.718 [-3.1 – 1.7]	-0.421 [-2.7 – 1.9]
	65–75 years	**-3.42** [-6.2 – -0.6]*	-2.27 [-5.0 – 0.4]
	76 + years	**-9.41** [-12.8 – -6.0]***	**-6.42** [-9.7 – -3.1]***
**Access**	18–29 years	-1.39 [-4.8 – 2.0]	0.228 [-3.1 – 3.5]
	30–45 years	ref.	ref.
	46–64 years	-1.91 [-4.7 – 0.8]	-1.52 [-4.1 – 1.1]
	65–75 years	**-5.12** [-8.3 – -1.9]**	**-3.82** [-6.9 – -0.8]**
	76 + years	**-13.03** [-16.9 – -9.2]***	**-9.46** [-13.2 – -5.7]***
**Understand**	18–29 years	-0.864 [-4.3 – 2.6]	1.09 [-2.2 – 4.4]
	30–45 years	ref.	ref.
	46–64 years	-1.36 [-4.2 – 1.4]	-0.916 [-3.6 – 1.7]
	65–75 years	**-6.49** [-9.7 – -3.3]***	**-4.90** [-8.0 – -1.8]**
	76 + years	**-11.57** [-15.5 – -7.6]***	**-7.60** [-11.4 – -3.8]***
**Appraise**	18–29 years	-3.71 [-7.8 – 0.4]	-2.14 [-6.2 – 1.9]
	30–45 years	ref.	ref.
	46–64 years	1.11 [-2.2 – 4.4]	1.36 [-3.3 – 4.1]
	65–75 years	-0.581 [-4.4 – 3.2]	0.396 [-8.6 – 0.5]
	76 + years	**-6.63** [-11.2 – -2.0]**	-4.04 [0.0 – 0.0]
**Apply**	18–29 years	2.27 [-1.0 – 5.5]	2.98 [-0.2 – 6.2]
	30–45 years	ref.	ref.
	46–64 years	-0.63 [-3.3 – 2.0]	-0.535 [-3.1 – 2.0]
	65–75 years	-1.38 [-4.4 – 1.7]	-0.643 [-3.6 – 2.3]
	76 + years	**-6.08** [-9.8 – -2.4]**	**-4.29** [-8.0 – -0.6]*

n = 1,759; ref.: reference group, CI: 95% confidence interval

^ adjusted for gender, educational level, literacy skills, social status, financial deprivation

*** p < .001, ** p < .01, * p < .05, bold numbers

### The role of social support for HL in different age groups

With regard to social support, HL is generally lower among people with poorer social support. The mean general HL score among people with poor social support is 54.7 while it is 61.6 for people with moderate and 65.6 for people with strong social support. This tendency holds true for all steps of information management (see supplementary Table 1).

**Fig. 2:**
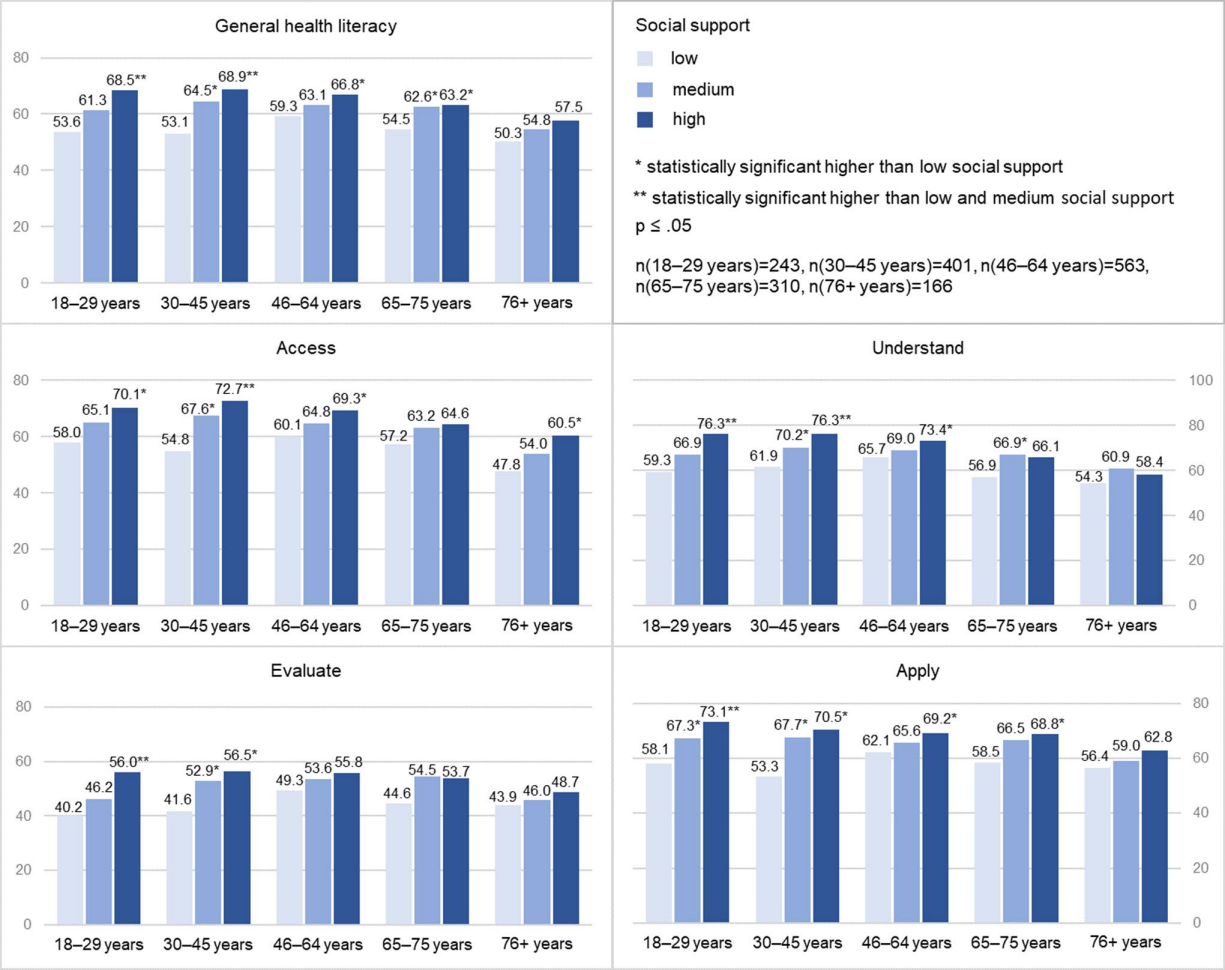
Mean scores of general HL and steps of information management by social support categories in age groups

Social support is pronounced quite similarly across age groups. While 11.7% of the respondents aged 18–29 years and 11.3% of those aged 30–45 years have poor social support, 19.5% of the respondents aged 76 years and older reported poor social support. The comparison of social support scores reveals a very small decrease in older age groups (see supplementary Table 2). Analysing the role of social support for HL in different age groups shows that the mean scores are lower among people with poor social support in all age groups and in all steps of information management (Fig. 2). In *accessing*, *appraising* and *applying* health information, scores increase with more social support among all age groups. In *understanding* information, scores only ascend continuously with more social support in the younger age groups (below 65 years) while they increase only from poor to moderate social support among respondents aged 65–75 years and do not increase with more social support among respondents aged 76 years and older. In general, the differences of mean scores by level of social support are larger among younger age groups and less pronounced in older age groups in all steps of information management. However, there are also considerable mean score differences by different social support levels in *accessing* information among respondents aged 76 years and older and *applying* information among respondents aged 65–75 years. All mean values with 95% confidence intervals can be found in supplementary Table 3.

Table 3 shows the coefficients of OLS regressions that control for confounding variables for the social support score (B_1_) and, for investigating the non-linear correlations described above, coefficients of strong in comparison to poor social support (B_2_). In the total sample, social support is positively linked to *general HL* and all steps of information management (p < .01) regardless of the confounding factors, i.e. HL is higher when social support is higher. The coefficients indicate that the bond is strongest for *accessing* (B_1_ = 1.40/B_2_ = 7.44) and lowest for *appraising* information (B_1_ = 0.909/B_2_ = 5.51). The standardized coefficients (see supplementary Table 4) reveal a small effect size of social support (β = 0.066 and β = 0.121 for the stated steps). Nevertheless, measured by the standardized coefficients its impact seems to be similar to that of social status (full regression results in supplementary able 4).

**Table 3:** OLS regression results of social support on general HL and steps of information management in total sample and age groups

Dependent variable (0–100)	Sample (unweighted)	Social support score (1–9) B_1_ [CI]	Strong vs. poor social support B_2_ [CI]
**General HL**	Total	**1.18** [0.7 – 1.6]***	**6.46** [3.6 – 9.3]***
	18–29 years	**1.53** [0.3 – 2.7]*	**9.50** [1.9 – 17.1]*
	30–45 years	1.59 [0.6 – 2.6]**	**11.07** [4.8 – 17.3]***
	46–64 years	0.71 [-0.1 – 1.5]	3.94 [-0.8 – 8.7]
	65–75 years	**1.25** [0.1 – 2.4]*	**8.35** [1.4 – 15.3]*
	76 + years	1.26 [-0.3 – 2.8]	0.41 [-9.3 – 10.1]
**Access**	Total	**1.40** [0.9 – 1.9]***	**7.44** [4.2 – 10.7]***
	18–29 years	1.02 [-0.3 – 2.3]	6.58 [-1.6 – 14.7]
	30–45 years	**1.90** [0.8 – 3.0]**	**13.93** [7.0 – 20.9]***
	46–64 years	**1.07** [0.2 – 2.0]*	**5.60** [0.2 – 11.0]*
	65–75 years	1.23 [-0.1 – 2.6]	6.85 [-1.2 – 14.9]
	76 + years	**1.94** [0.1 – 3.8]*	4.13 [-7.2 – 15.5]
**Understand**	Total	**1.10** [0.6 – 1.6]***	**6.10** [2.8 – 9.4]***
	18–29 years	**2.02** [0.6 – 3.5]**	7.76 [-1.5 – 17.1]
	30–45 years	**1.83** [0.7 – 3.0]**	**10.98** [3.8 – 18.2]**
	46–64 years	0.45 [-0.5 – 1.4]	3.83 [-1.6 – 9.3]
	65–75 years	1.00 [-0.3 – 2.3]	**8.84** [0.9 – 16.8]*
	76 + years	0.49 [-1.2 – 2.2]	-2.71 [-13.3 – 7.9]
**Appraise**	Total	**0.91** [0.3 – 1.6]**	**5.51** [1.6 – 9.5]**
	18–29 years	**1.82** [0.1 – 3.6]*	11.00 [-0.3 – 22.3]
	30–45 years	0.95 [-0.5 – 2.4]	8.31 [-0.7 – 17.3]
	46–64 years	0.52 [-0.6 – 1.6]	2.52 [-4.0 – 9.1]
	65–75 years	1.08 [-0.5 – 2.6]	**9.60** [0.3 – 18.9]*
	76 + years	1.09 [-1.0 – 3.2]	-1.03 [-13.9 – 11.9]
**Apply**	Total	**1.30** [0.8 – 1.8]***	**6.75** [3.6 – 9.9]***
	18–29 years	1.27 [0.0 – 2.6]	**12.98** [4.9 – 21.1]**
	30–45 years	**1.68** [0.5 – 2.8]**	**10.95** [3.8 – 18.1]**
	46–64 years	0.74 [-0.1 – 1.6]	3.71 [-1.5 – 8.9]
	65–75 years	**1.70** [0.5 – 2.9]**	**8.31** [0.7 – 15.9]*
	76 + years	1.41 [-0.3 – 3.1]	0.78 [-9.6 – 11.2]

Unstandardized coefficient (B) adjusted for gender, educational level, literacy skills, social status, financial deprivation

*** p < .001, ** p < .01, * p < .05, bold numbers; CI: 95% confidence interval

n(Total) = 1,700, n(18–29 years) = 246, n(30–45 years) = 399, n(46–64 years) = 563, n(65–75 years) = 309, n(76 + years) = 169

Furthermore, the regression results support most findings shown in Fig. 2. The association of HL and social support is not the same in different age groups. Generally speaking, the link is stronger in younger age groups (below 46 years) as well as for young-old people (65–75 years) and weaker for old middle-aged adults (46–64 years) and old-old people but with the following exceptions. After adjusting for confounders, social support is *not* statistically significant connected with *accessing* information in the youngest group (B_1_ = 1.02/B_2_ = 6.58, p > .05) and young-old people (B_1_ = 1.23/B_2_ = 6.85, p > .05) as well as *appraising* information among 30–45-year-olds (B_1_ = 0.946/B_2_ = 8.31, p > .05), indicating that an essential part of the bivariate relation is influenced by common determinants. As already shown in the graphical analyses, the link for *accessing* information is relatively strong in the oldest group (B_1_ = 1.94, p < .05) though the coefficient of strong in comparison to poor social support is rather small and not statistically significant (B_2_ = 4.13, p > .05). However, it should be noted that the effect sizes of social support are rather small even in age groups with the strongest relations (see supplementary Table 4 for standardized coefficients and other values). Still, in some cases the effect size is bigger than those of other established factors, e.g. for the youngest adults, social support (ß=0.177) seems to be more relevant for *understanding* health information than their educational level (ß=0.152) or social status (ß=0.031, p > .05).

## Discussion

Our study investigated the role of social support for comprehensive HL in different age groups, specifically, the aspect of informational support that social contacts can provide and thus help with the challenges of health information management [16]. This contributes to the debate on the importance of the social context in which HL is formed and used [12, 13, 15]. Our study focuses on individuals but uses a relational concept of HL which includes the health information environment and therefore is on the intersection of HL as an individual skill and a social practice [13].

The results show that comprehensive HL is positively associated with social support in general. This is in line with the little prior quantitative research studying this relation in different countries and with different measurements for both constructs, e.g. among adolescents and adults in Germany [19, 21, 22] and students in Pakistan [23]. However, the role of social support on comprehensive HL has not been analysed across the life span so far. We addressed this gap by analysing HL in different age groups. In general, our findings show that the association of HL and social support is not the same in different age groups and thus possibly not constant across the life span. Among young and young middle-aged adults (persons aged 18–45 years) as well as young-old people (65–75 years) the relation of social support and HL is stronger than among old middle-aged (46–64 years) and old-old people (76 and more years). That is, they seem to be more responsive and receptive to informational support from their social network. Furthermore, there is variance of this association within age groups in the steps of information management.

We could observe a tendency of slightly lower HL among young adults compared to middle-aged adults in our data which makes them a vulnerable group in regard of managing health information. In this early phase of adult life, individuals have to learn to take more responsibility for their own health as they become more independent from parents or other caregivers. They have to learn to make (informed) decisions about their health and thus are still in a learning phase regarding their health information management. They also face relatively few health problems [52] and accordingly have to deal with fewer health information and therefore might lack the experience in managing health information which might result in low HL. While low HL among young adults was also found in samples from Albania [28], Denmark, Israel and Slovakia [43] and is also supported by findings showing low HL among adolescents [22, 53], this has not been observed in most countries of the HLS_19_ [43], other international studies [28, 41, 54] or other samples from Germany [29, 55]. We assume that different study populations and especially different conditions of the health (information) environment specifically for young people (e.g. integration of HL in school curricula) might lead to these contradictory findings. In Finland, for example, where children and adolescents are trained in schools, HL is higher among young adults [56]. In Germany, efforts to integrate HL in school curricula have just started [57]. The results also indicate, that it may be relevant to further assess and compare HL throughout the socialisation process of young people (including childhood, adolescence and young adulthood), instead of an orientation by formal age of majority. It might also be interesting to relate HL to specific aspects of health relevant for this phase of life, such as sexual health.

In this regard, it seems plausible that younger adults who first have to learn to manage health information on their own still rely on their primary source of help and social support – their family [58, 59] and thus are more responsive to social support. This can be seen as part of the socialization process and supports the assumption that HL is a social practice. Qualitative studies emphasize the relevance of social support among these age groups [25, 60]. Additionally, by comparing the steps of information management our results indicate that young adults seem to be particularly more responsive to social support for *understanding, appraising* and *applying* but not for *accessing* information. An explanation might be that this digitally native generation does not need help with finding information because they are easily available on the internet. But for cognitively more challenging tasks of understanding and appraising the information this age group could profit from exchange with more experienced persons of their social network. *Appraising* health information is perceived as particularly challenging by youngest adults – the gap to middle aged adults is largest in this step. This may be explained with the necessity to learn the management of health information and gain knowledge about criteria for good and reliable health information also by experience to become health literate. Additionally, the difficulties in appraising health information might be driven by their affinity to access health information from the internet where they find a vast body of information from different sources that is more challenging to be classified as appropriate and reliable [61]. With our findings we show that young adults need to be seen as a potentially vulnerable group with difficulties in health information management and therefore should be targeted by HL interventions.

Generally, individuals in middle adulthood (around 30 to 64 years) have rather high HL. This is supported by some other international studies [9, 28, 41, 43, 55]. This is plausible as they have increasing experience in managing their health information. In addition, they – especially women – have increasing responsibility for other people’s health and can be called “information managers”, “surrogate seekers” or “knowledge brokers” e.g. for children, partners or older relatives like parents [62–64]. Thus, they have a lot of experience in managing health information which might increase their HL.

Interestingly, although these age groups are not particularly vulnerable for low HL, the association of HL with social support is still quite high, especially for people aged 30 to 45. A reason might be that people in this age group have broad social networks, e.g. through their children and work, and thus can easily use but also *give* informational support. Therefore, sharing good information or information sources with contacts who are in the same responsible position might be more common and relevant for overcoming the obstacles that come with information management in this phase of life. Although older middle-aged adults (46–64 years) have a very similar HL as young middle-aged adults, HL and social support are not as strongly related in this age group.

Looking at the later phases in the life span (65 + years), our study shows that only the oldest respondents (76 years and older) have lower HL compared to younger age groups throughout the information management process. Similar patterns could be found in country samples of the first European HL study (HLS-EU) [4], the first nationwide HL study in Germany [47] and in a Lithuanian sample [32]. In contrast, results in other countries of the HLS-EU [4] and the HLS_19_ [43] as well as in two German samples [36, 55] do not show these differences in HL between young-old and old-old people. We suspect several possible explanations for these mixed findings. First, it can be ascribed to different study populations, e.g. patients or socioeconomically advantaged persons, in which different mechanisms occur. Second, in some contexts, e.g. countries or populations young-old and old-old people might not differ much regarding their vulnerability for low HL, i.e. young-old people might have similar socio-economic or health-related disadvantages as old-old people. Third, since these studies used relational concepts of HL that include the conditions of the health (information) environment, the differences may be due to variations in those conditions. That is, those environments in which young and old-old people have the same HL might provide more helpful options for the challenges old-old persons have to face with health information management. However, further research is necessary to investigate this; especially as the late phase of life is often not differentiated [9, 28, 30, 33, 38, 39, 54].

However, in contrast to the “vulnerability hypothesis”, the relation of social support and HL is stronger for young-old people and barely existent for old-old people. A possible explanation is the beginning of a new phase of life, for most people particularly the end of employment and their professional activity which results in new requirements regarding health management which might lead to the increased relation with social support. In addition, young-old persons might be more socially engaged and thus have easier access to social support. For old-old persons, the resources and abilities of social contacts may not be sufficient to help with informational support regarding the often complex health or illness situations a lot of them have to manage. In addition, they tend to have less social support [21, 45]. Additionally, studies showed that old-aged people state health professionals as (primary) source of and help to manage health information more often than younger people [65–67], which might not be considered as social support. Therefore, they might have little need to discuss health information with other people in their social network. It is noteworthy though, that some old-old persons might need social support initially for finding the appropriate health professional in a complex health system and its organisations [68, 69]. Moreover, also the communication with health professionals can be challenging which increases the need for social support from family and friends in the form of so-called informal health advocates [70]. A further investigation on the role of social support for other HL requirements, that is navigational and communicative HL [68, 71, 72], among old-old people is recommended. Nevertheless, old-old people who are more vulnerable for low HL, e.g. chronically ill, less educated or face language barriers, might still benefit from social support regarding their HL.

### Limitations

Finally, possible limitations should be discussed. First of all, HL was measured using a perception-based instrument (HLS_19_-Q47). Although the instrument is widely used in HL research, there are some aspects to consider, such as overestimation of one’s competence in self-assessment [73, 74]. A detailed reflection on the instrument has already been described [75, 76].

We performed a cross-sectional study and thus we cannot answer whether HL decreases over the course of life. However, analysing different age groups in detail provided interesting insights into challenges in different phases of life.

The sample of this study is representative of the adult population regarding gender in combination with age group and educational level. However, the single age groups are not representative for other demographic and socioeconomic characteristics. This limits the generalisability of the results. Though, an advantage of this study is the survey inclusion of a broad range of the population especially in old age by conducting personal oral interviews instead of web-based data collection which would lead to more self-selection bias in data.

Additionally, social support in our study sample is higher than in two other German study samples [21, 45]. This might be a result of the sampling strategy that is based on recruitment of participants through the interviewers which is not a completely random sampling strategy as in the other studies. Therefore, socially more isolated persons had a lesser chance to be included in the study. The amount of social support is similar in all age groups with a tendency of slightly lower social support in older age groups. This finding is consistent with the two German studies [21, 45].

Furthermore, the instrument used for measuring social support needs to be reflected. The Oslo 3 Social Support Scale measures social support with three rather general questions. However, the instrument is a brief and valid option to measure social support [45]. Future research could focus on the informational aspect and/or health-related forms of social support. Additionally, qualitative studies might add relevant information to the nuances of the relationship of social support and HL [25]. This also applies to the finding of non-linear associations between social support and HL in some age groups which should be considered in future studies. Moreover, there are possibly other factors influencing the associations that were not included in our study but should be investigated in future research.

## Conclusion

Our results indicate that HL is a social practice [24] and supports the demand to include the social context into the understanding as well as the improvement of HL [13, 14]. Although, social support and HL are positively related, this association is not the same for all adult age groups and thus possibly across the life span.

Our study demonstrates that it is necessary to differentiate between subgroups and situational requirements as social support seems to be more beneficial for the HL of some than of others. Although specifically old-old people are strongly challenged by health information management and have on average the lowest HL of all adults this can barely be compensated by more social support. This is not the case for young-old people who have higher HL and are even similarly challenged by some information management steps as younger adults, namely by appraising and applying. In this life phase people do indeed seem to profit from social support. With this found distinction, we advise that the late life phase needs to be further stratified to appropriately describe the needs and obstacles old-aged persons have regarding their HL. This approach has long been used in gerontological research and will be even more important in the next decades given the ageing of societies and the increase in life expectancy. In addition, our results suggest that young adults are challenged by health information management as well. In contrast to the oldest group, they can strongly profit from social support.

Additionally, our study shows that the relation of comprehensive HL and age is not linear in the German population. However, as HL is relatively low in all adult age groups, interventions for everyone need to be initiated. Additionally, a focus on interventions to make appraising information less challenging would be beneficial. Furthermore, our study reveals special needs for the oldest age group especially regarding the access and understanding of health information. Overall, due to increasing cognitive impairment, strategies for old-aged people should focus on external conditions instead of skill trainings, i.e. increase the usability of health information, health organisations and the health system as a whole [2, 77, 78]. In contrast, the youngest adults lower HL and particularly challenges with appraising health information can further be addressed by skill trainings. Summarized, interventions should not simply take older age into account, but consider the obstacles and needs in different life phases and different steps of health information management. Thus, target group specific services and programs are needed.

### Electronic supplementary material

Below is the link to the electronic supplementary material.

Supplementary Material 1

## Data Availability

The datasets used and/or analysed during the current study are available from the corresponding author on reasonable request.
