# Supplementary material for: Health literacy and the role of social support in different age groups: results of a German cross-sectional survey
Source: BMC Public Health. 2023 Nov 16;23:2259. doi: 10.1186/s12889-023-17145-x (PMC10652531; doi:10.1186/s12889-023-17145-x)
Supplement: Supplementary file 1 — Supplementary Material 1 [file 12889_2023_17145_MOESM1_ESM.docx]

***Supplementary Table 1:*** *Score means and [95%-confidence intervals] in total sample and age groups, and in social support categories*

|  | **General HL** | **Access** | **Understand** | **Appraise** | **Apply** |
| --- | --- | --- | --- | --- | --- |
| **Total**^ | **61.8** [60.9 – 62.7] | **63.7** [62.7 – 64.7] | **67.4** [66.4 – 68.3] | **51.3** [50.1 – 52.4] | **65.4** [64.5 – 66.3] |
| **Age group** |  |  |  |  |  |
| 18‒29 years | **62.1** [59.9 – 64.3] | **64.8** [62.4 – 67.2] | **68.7** [66.1 – 71.3] | **48.5** [45.6 – 51.4] | **67.2** [65.0 – 69.5] |
| 30‒45 years | **64.7** [63.0 – 66.4] | **67.9** [66.0 – 69.7] | **71.4** [69.5 – 73.4] | **53.0** [50.6 – 55.3] | **66.9** [65.0 – 68.8] |
| 46‒64 years | **63.9** [62.4 – 65.3] | **65.7** [64.0 – 67.4] | **70.1** [68.4 – 71.7] | **54.1** [52.1 – 56.0] | **66.3** [64.7 – 67.8] |
| 65‒75 years | **61.7** [59.6 – 63.8] | **63.1** [60.7 – 65.4] | **65.0** [62.7 – 67.4] | **53.0** [50.4 – 55.7] | **65.9** [63.7 – 68.1] |
| 76+ years | **54.4** [51.5 – 57.3] | **54.5** [51.1 – 57.9] | **58.2** [55.0 – 61.4] | **46.1** [42.4 – 49.7] | **59.6** [56.6 – 62.5] |
| **Social support**^ |  |  |  |  |  |
| Low | **54.7** [52.4 – 57.1] | **55.5** [52.8 – 58.2] | **60.9** [58.2 – 63.7] | **44.9** [41.9 – 47.8] | **57.8** [55.2 – 60.3] |
| Medium | **61.6** [60.3 – 62.8] | **63.5** [62.1 – 65.0] | **67.0** [65.5 – 68.4] | **50.9** [49.2 – 52.5] | **65.3** [64.0 – 66.6] |
| High | **65.6** [64.3 – 67.0] | **68.1** [66.6 – 69.7] | **71.1** [69.6 – 72.7] | **54.4** [52.6 – 56.3] | **69.4** [68.0 – 70.9] |

*^ weighted data*

***Supplementary Table 2:*** *Social support in age groups*

| **Age group** | **Low** % (n) | **Medium** % (n) | **High** % (n) | **Mean score social support** (range 4‒14) [95 % confidence interval] |
| --- | --- | --- | --- | --- |
| 18‒29 years | 11.7 (37) | 48.9 (154) | 39.4 (124) | 10.9 [10.7 – 11.1] |
| 30‒45 years | 11.3 (54) | 48.1 (229) | 40.5 (193) | 10.8 [10.7 – 11.0] |
| 46‒64 years | 16.0 (105) | 49.0 (321) | 35.0 (229) | 10.6 [10.4 – 10.7] |
| 65‒75 years | 13.9 (52) | 50.8 (190) | 35.3 (132) | 10.6 [10.3 – 10.8] |
| 76+ years | 19.5 (43) | 47.5 (105) | 33.0 (73) | 10.4 [10.1 – 10.7] |
|  | Chi²=14.4 (p=.072) |  |  |  |

***Supplementary Table 3:*** *Score means and [95%-confidence intervals] in social support categories in age groups*

| **Dependent  variable**  (score 0‒100) | **Sample** (unweighted) | **Low** | **Medium** | **High** |
| --- | --- | --- | --- | --- |
| **General HL** | 18‒29 years | 53.6 [47.1 ‒ 60.1] | 61.3 [58.2 ‒ 64.5] | 68.5 [65.3 ‒ 71.6] |
|  | 30‒45 years | 53.1 [47.3 ‒ 58.9] | 64.5 [62.0 ‒ 67.1] | 68.9 [66.5 ‒ 71.2] |
|  | 46‒64 years | 59.3 [55.7 ‒ 62.9] | 63.1 [60.9 ‒ 65.2] | 66.8 [64.4 ‒ 69.2] |
|  | 65‒75 years | 54.5 [48.2 ‒ 60.9] | 62.6 [59.7 ‒ 65.6] | 63.2 [59.9 ‒ 66.5] |
|  | 76+ years | 50.3 [43.9 ‒ 56.8] | 54.8 [50.5 ‒ 59.0] | 57.5 [52.2 ‒ 62.9] |
| **Access** | 18‒29 years | 58.0 [49.9 ‒ 66.1] | 65.1 [61.7 ‒ 68.5] | 70.1 [66.8 ‒ 73.5] |
|  | 30‒45 years | 54.8 [48.4 ‒ 61.2] | 67.6 [65.0 ‒ 70.3] | 72.7 [69.9 ‒ 75.4] |
|  | 46‒64 years | 60.1 [55.8 ‒ 64.4] | 64.8 [62.3 ‒ 67.2] | 69.3 [66.5 ‒ 72.2] |
|  | 65‒75 years | 57.2 [50.2 ‒ 64.2] | 63.2 [59.6 ‒ 66.7] | 64.6 [60.9 ‒ 68.3] |
|  | 76+ years | 47.8 [40.3 ‒ 55.3] | 54.0 [49.0 ‒ 59.0] | 60.5 [54.3 ‒ 66.8] |
| **Understand** | 18‒29 years | 59.3 [51.3 ‒ 67.3] | 66.9 [63.2 ‒ 70.6] | 76.3 [72.6 ‒ 80.1] |
|  | 30‒45 years | 61.9 [55.7 ‒ 68.0] | 70.2 [67.3 ‒ 73.2] | 76.3 [73.7 ‒ 79.0] |
|  | 46‒64 years | 65.7 [61.4 ‒ 70.1] | 69.0 [66.5 ‒ 71.5] | 73.4 [70.6 ‒ 76.2] |
|  | 65‒75 years | 56.9 [49.6 ‒ 64.3] | 66.9 [63.6 ‒ 70.3] | 66.1 [62.4 ‒ 69.8] |
|  | 76+ years | 54.3 [46.3 ‒ 62.2] | 60.9 [56.1 ‒ 65.7] | 58.4 [52.8 ‒ 63.9] |
| **Appraise** | 18‒29 years | 40.2 [31.6 ‒ 48.8] | 46.2 [42.0 ‒ 50.3] | 56.0 [51.3 ‒ 60.7] |
|  | 30‒45 years | 41.6 [34.1 ‒ 49.1] | 52.9 [49.3 ‒ 56.5] | 56.5 [53.1 ‒ 59.9] |
|  | 46‒64 years | 49.3 [44.5 ‒ 54.1] | 53.6 [50.7 ‒ 56.5] | 55.8 [52.5 ‒ 59.0] |
|  | 65‒75 years | 44.6 [36.7 ‒ 52.4] | 54.5 [50.7 ‒ 58.3] | 53.7 [49.4 ‒ 58.0] |
|  | 76+ years | 43.9 [36.3 ‒ 51.6] | 46.0 [40.8 ‒ 51.2] | 48.7 [41.6 ‒ 55.9] |
| **Apply** | 18‒29 years | 58.1 [51.1 ‒ 65.1] | 67.3 [64.2 ‒ 70.4] | 73.1 [69.7 ‒ 76.5] |
|  | 30‒45 years | 53.3 [46.8 ‒ 59.9] | 67.7 [65.0 ‒ 70.4] | 70.5 [67.7 ‒ 73.2] |
|  | 46‒64 years | 62.1 [58.0 ‒ 66.2] | 65.6 [63.3 ‒ 67.9] | 69.2 [66.6 ‒ 71.7] |
|  | 65‒75 years | 58.5 [51.3 ‒ 65.6] | 66.5 [63.4 ‒ 69.6] | 68.8 [65.3 ‒ 72.3] |
|  | 76+ years | 56.4 [49.2 ‒ 63.5] | 59.0 [54.6 ‒ 63.4] | 62.8 [57.6 ‒ 67.9] |
| n(18‒29 years)=243, n(30‒45 years)=401, n(46‒64 years)=563, n(65‒75 years)=310, n(76+ years)=166 | | | | |

***Supplementary Table 4:*** *Full regression results*

|  |  | **Total sample** | | | | **18‒29 years** | | | | **30‒45 years** | | | | **46‒64 years** | | | | **65‒75 years** | | | | **76 and more years** | | | |
| --- | --- | --- | --- | --- | --- | --- | --- | --- | --- | --- | --- | --- | --- | --- | --- | --- | --- | --- | --- | --- | --- | --- | --- | --- | --- |
|  |  | B | se | p | ß | B | se | p | ß | B | se | p | ß | B | se | p | ß | B | se | p | ß | B | se | p | ß |
| **General health literacy** | Social support | 1.177 | 0.240 | <.001 | 0.116 | 1.525 | 0.605 | .012 | 0.163 | 1.585 | 0.514 | .002 | 0.153 | 0.705 | 0.406 | .083 | 0.072 | 1.248 | 0.582 | .033 | 0.119 | 1.256 | 0.794 | .116 | 0.118 |
|  | Gender | 1.974 | 0.894 | .027 | 0.051 | 2.266 | 2.217 | .308 | 0.063 | 1.594 | 1.805 | .378 | 0.043 | 3.770 | 1.542 | .015 | 0.099 | 2.615 | 2.215 | .239 | 0.066 | -1.453 | 3.304 | .661 | -0.033 |
|  | Education | 1.825 | 0.384 | <.001 | 0.120 | 2.477 | 1.027 | .017 | 0.152 | 1.647 | 0.786 | .037 | 0.114 | 1.198 | 0.68 | .079 | 0.077 | 1.572 | 0.902 | .082 | 0.109 | 3.043 | 1.444 | .037 | 0.175 |
|  | Literacy skills | 1.580 | 0.287 | <.001 | 0.129 | 1.584 | 0.742 | .034 | 0.134 | 0.783 | 0.657 | .234 | 0.059 | 2.008 | 0.522 | <.001 | 0.157 | 0.236 | 0.648 | .716 | 0.021 | 2.397 | 0.909 | .009 | 0.193 |
|  | Social status | 1.473 | 0.341 | <.001 | 0.117 | 0.618 | 0.839 | .462 | 0.050 | 1.151 | 0.734 | .118 | 0.092 | 1.556 | 0.573 | .007 | 0.132 | 2.360 | 0.845 | .006 | 0.180 | 1.390 | 1.186 | .243 | 0.104 |
|  | Financial depriva. | -3.695 | 1.331 | .006 | -0.071 | -2.033 | 2.959 | .493 | -0.045 | -4.76 | 2.817 | .092 | -0.089 | -3.859 | 2.496 | .123 | -0.071 | -2.772 | 3.131 | .377 | -0.054 | -5.470 | 4.217 | .196 | -0.103 |
| **Access health information** | Social support | 1.397 | 0.274 | <.001 | 0.121 | 1.022 | 0.648 | .116 | 0.102 | 1.899 | 0.574 | .001 | 0.166 | 1.066 | 0.466 | .023 | 0.095 | 1.243 | 0.669 | .064 | 0.103 | 1.941 | 0.924 | .037 | 0.151 |
|  | Gender | 0.149 | 1.021 | .884 | 0.003 | 1.044 | 2.375 | .661 | 0.027 | -1.278 | 2.014 | .526 | -0.031 | 3.245 | 1.770 | .067 | 0.074 | -0.062 | 2.547 | .981 | -0.001 | -3.547 | 3.843 | .357 | -0.067 |
|  | Education | 2.256 | 0.438 | <.001 | 0.130 | 2.907 | 1.100 | .009 | 0.167 | 2.043 | 0.877 | .020 | 0.128 | 1.079 | 0.780 | .167 | 0.061 | 2.418 | 1.038 | .020 | 0.145 | 4.367 | 1.679 | .010 | 0.210 |
|  | Literacy skills | 1.905 | 0.328 | <.001 | 0.136 | 1.886 | 0.795 | .019 | 0.149 | 0.939 | 0.733 | .201 | 0.064 | 2.291 | 0.599 | <.001 | 0.156 | 0.343 | 0.745 | .645 | 0.026 | 2.722 | 1.057 | .011 | 0.183 |
|  | Social status | 1.687 | 0.389 | <.001 | 0.117 | 1.421 | 0.899 | .115 | 0.108 | 0.512 | 0.819 | .532 | 0.037 | 2.072 | 0.658 | .002 | 0.152 | 2.866 | 0.971 | .003 | 0.190 | 1.348 | 1.379 | .330 | 0.084 |
|  | Financial depriva. | -3.309 | 1.520 | .030 | -0.055 | -1.102 | 3.169 | .728 | -0.023 | -3.843 | 3.142 | .222 | -0.065 | -4.103 | 2.865 | .153 | -0.065 | 0.653 | 3.600 | .856 | 0.011 | -10.23 | 4.905 | .039 | -0.160 |
| **Understand health information** | Social support | 1.095 | 0.278 | <.001 | 0.093 | 2.020 | 0.737 | .007 | 0.177 | 1.831 | 0.587 | .002 | 0.154 | 0.446 | 0.471 | .344 | 0.039 | 1.002 | 0.668 | .135 | 0.084 | 0.494 | 0.874 | .573 | 0.042 |
|  | Gender | 2.374 | 1.036 | .022 | 0.053 | 2.134 | 2.701 | .430 | 0.049 | 2.407 | 2.061 | .243 | 0.056 | 4.046 | 1.788 | .024 | 0.091 | 3.968 | 2.543 | .120 | 0.088 | -2.600 | 3.638 | .476 | -0.053 |
|  | Education | 2.749 | 0.444 | <.001 | 0.156 | 3.034 | 1.251 | .016 | 0.152 | 2.482 | 0.897 | .006 | 0.150 | 2.847 | 0.788 | <.001 | 0.157 | 2.419 | 1.036 | .020 | 0.147 | 3.185 | 1.590 | .047 | 0.168 |
|  | Literacy skills | 2.307 | 0.333 | <.001 | 0.163 | 2.459 | 0.904 | .007 | 0.17 | 1.311 | 0.750 | .081 | 0.086 | 2.641 | 0.605 | <.001 | 0.177 | 0.816 | 0.744 | .274 | 0.063 | 3.148 | 1.001 | .002 | 0.232 |
|  | Social status | 1.295 | 0.395 | .001 | 0.089 | 0.465 | 1.022 | .650 | 0.031 | 0.921 | 0.838 | .272 | 0.064 | 1.308 | 0.665 | .050 | 0.095 | 1.987 | 0.970 | .041 | 0.133 | 1.781 | 1.306 | .175 | 0.121 |
|  | Financial depriva. | -2.835 | 1.542 | .066 | -0.047 | -0.762 | 3.604 | .833 | -0.014 | -4.523 | 3.215 | .160 | -0.074 | -3.428 | 2.894 | .237 | -0.054 | -1.369 | 3.594 | .704 | -0.023 | -2.824 | 4.644 | .544 | -0.048 |
| **Appraise health information** | Social support | 0.909 | 0.332 | .006 | 0.066 | 1.823 | 0.900 | .044 | 0.133 | 0.946 | 0.735 | .199 | 0.065 | 0.523 | 0.563 | .353 | 0.04 | 1.078 | 0.781 | .169 | 0.078 | 1.091 | 1.058 | .304 | 0.081 |
|  | Gender | 4.880 | 1.241 | <.001 | 0.093 | 7.196 | 3.298 | .030 | 0.137 | 4.589 | 2.579 | .076 | 0.087 | 5.297 | 2.138 | .014 | 0.103 | 5.168 | 2.973 | .083 | 0.100 | 0.249 | 4.404 | .955 | 0.004 |
|  | Education | 2.008 | 0.532 | <.001 | 0.098 | 3.324 | 1.527 | .030 | 0.139 | 2.353 | 1.123 | .037 | 0.116 | 1.165 | 0.943 | .217 | 0.056 | 1.249 | 1.211 | .303 | 0.066 | 1.476 | 1.924 | .444 | 0.068 |
|  | Literacy skills | 1.261 | 0.399 | .002 | 0.076 | 1.510 | 1.104 | .173 | 0.087 | 0.882 | 0.939 | .348 | 0.047 | 1.769 | 0.723 | .015 | 0.103 | -0.428 | 0.870 | .623 | -0.029 | 2.312 | 1.211 | .058 | 0.148 |
|  | Social status | 1.389 | 0.473 | .003 | 0.082 | 0.100 | 1.248 | .936 | 0.006 | 1.546 | 1.049 | .141 | 0.088 | 1.318 | 0.795 | .098 | 0.083 | 2.306 | 1.134 | .043 | 0.134 | 1.361 | 1.581 | .390 | 0.081 |
|  | Financial depriva. | -4.011 | 1.847 | .030 | -0.057 | -2.458 | 4.401 | .577 | -0.037 | -6.479 | 4.024 | .108 | -0.086 | -2.808 | 3.461 | .418 | -0.038 | -4.702 | 4.202 | .264 | -0.070 | -3.101 | 5.621 | .582 | -0.046 |
| **Apply health information** | Social support | 1.295 | 0.264 | <.001 | 0.119 | 1.270 | 0.650 | .052 | 0.132 | 1.678 | 0.588 | .005 | 0.145 | 0.741 | 0.450 | .100 | 0.07 | 1.699 | 0.632 | .008 | 0.150 | 1.407 | 0.849 | .099 | 0.127 |
|  | Gender | 0.554 | 0.987 | .575 | 0.013 | -1.534 | 2.383 | .520 | -0.041 | 0.949 | 2.066 | .646 | 0.023 | 2.368 | 1.710 | .167 | 0.058 | 1.674 | 2.405 | .487 | 0.039 | 0.481 | 3.532 | .892 | 0.010 |
|  | Education | 0.180 | 0.423 | .670 | 0.011 | 0.468 | 1.103 | .672 | 0.028 | -0.427 | 0.899 | .635 | -0.027 | -0.309 | 0.754 | .682 | -0.018 | 0.100 | 0.980 | .919 | 0.006 | 3.101 | 1.543 | .046 | 0.173 |
|  | Literacy skills | 0.815 | 0.317 | .010 | 0.062 | 0.441 | 0.798 | .581 | 0.036 | -0.033 | 0.752 | .966 | -0.002 | 1.283 | 0.578 | .027 | 0.093 | 0.254 | 0.704 | .719 | 0.021 | 1.335 | 0.972 | .171 | 0.104 |
|  | Social status | 1.506 | 0.376 | <.001 | 0.112 | 0.412 | 0.902 | .648 | 0.033 | 1.728 | 0.840 | .040 | 0.123 | 1.472 | 0.636 | .021 | 0.115 | 2.194 | 0.917 | .017 | 0.155 | 1.047 | 1.268 | .410 | 0.076 |
|  | Financial depriva. | -4.679 | 1.469 | .001 | -0.083 | -3.988 | 3.180 | .211 | -0.086 | -4.133 | 3.222 | .200 | -0.069 | -5.093 | 2.768 | .066 | -0.087 | -6.151 | 3.399 | .071 | -0.111 | -5.362 | 4.508 | .236 | -0.097 |

Social support: 1‒9, Gender: 0=men 1=women, Education: 1‒6, Literacy skills: 1‒6, Social status: 1‒10, Financial deprivation: 0=no 1=yes (2 or all 3 questions regarding difficulties in paying for medications, medical treatments, and monthly bills answered with “(very) difficult”)

n(Total)=1,700, n(18-29)=246, n(30-45)=399, n(46-64)=563, n(65-75)=309, n(76+)=169 B: unstandardized coefficient, se: standard error, p: p-value, ß: standardized coefficient
